# Genome Editing of Pigs for Agriculture and Biomedicine

**DOI:** 10.3389/fgene.2018.00360

**Published:** 2018-09-04

**Authors:** Huaqiang Yang, Zhenfang Wu

**Affiliations:** National Engineering Research Center for Breeding Swine Industry, College of Animal Science, South China Agricultural University, Guangzhou, China

**Keywords:** pig, genome editing, ZFN, TALEN, CRISPR/Cas9, disease model, xenotransplantation, breeding

## Abstract

Pigs serve as an important agricultural resource and animal model in biomedical studies. Efficient and precise modification of pig genome by using recently developed gene editing tools has significantly broadened the application of pig models in various research areas. The three types of site-specific nucleases, namely, zinc-finger nucleases, transcription activator-like effector nucleases, and clustered regularly interspaced short palindromic repeats (CRISPR)/CRISPR-associated protein, are the main gene editing tools that can efficiently introduce predetermined modifications, including knockouts and knockins, into the pig genome. These modifications can confer desired phenotypes to pigs to improve production traits, such as optimal meat production, enhanced feed digestibility, and disease resistance. Besides, given their genetic, anatomic, and physiologic similarities to humans, pigs can also be modified to model human diseases or to serve as an organ source for xenotransplantation to save human lives. To date, many genetically modified pig models with agricultural or biomedical values have been established by using gene editing tools. These pig models are expected to accelerate research progress in related fields and benefit humans.

## Introduction

Pigs hold great promise in agriculture and biomedicine. As an important meat source, domestic pigs provide the most commonly consumed meat worldwide. Through selective breeding, humans produce pigs that harbor desired characteristics for agriculture, albeit the selection is a long and slow process (Ruan et al., 2017). However, the process can now be substantially revolutionized and accelerated through genetic modification, including random transgenesis and gene knockouts and knockins (Gaj et al., 2013; Garas et al., 2015). With the improved efficiency of genetic modification, pig genome modification can confer any desired, predetermined genetic changes, which would take years to be realized in traditional selective breeding. Numerous economically significant characteristics, such as increased meat production (Qian et al., 2015; Wang et al., 2015, 2017b; Bi et al., 2016; Rao et al., 2016), reduced fat deposition (Zheng et al., 2017), or enhanced disease resistance (Whitworth et al., 2016; Burkard et al., 2017; Wells et al., 2017; Yang et al., 2018), have been achieved simply and efficiently through genetic modification in pigs, which can be used as valuable breeding materials to advance pig production. In biomedical research, pigs serve as an important large animal model given their advantages over other models. Compared with rodent models, pigs share a higher similarity to human beings in terms of body/organ size, lifespan, anatomy, physiology, and metabolic profile. Compared with non-human primates, pigs have low-cost and mature embryonic manipulation techniques. Pigs can be modified to carry the same gene mutation found in humans to replicate inherited diseases (Perleberg et al., 2018), or offer organs with minimal transplant rejection during xenotransplantation (Hryhorowicz et al., 2017). Pigs bridge the gap between humans and the heavily used small rodent models to favor biomedical research ranging from basic science to translational medicine.

**FIGURE 1:**
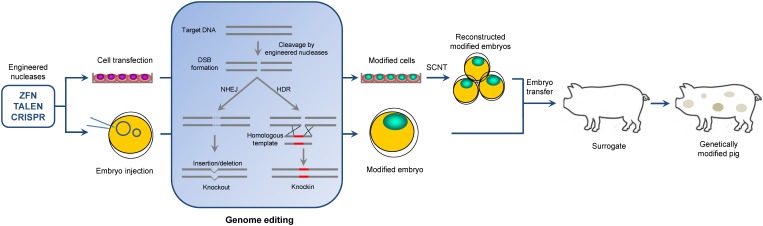
Overview of genome editing in the pig. Two ways to generate genome-edited pigs are shown: embryo injection of engineered nucleases and somatic cell nuclear transfer. In genome editing process, engineered nucleases induce a DSB at the target site. Subsequent DNA-repair by NHEJ and HDR pathways can introduce site-specific knockout and knockin, respectively, in the pig genome.

However, production of gene-targeted mammals other than mice remained difficult until the late 2000s because the traditional gene targeting technique developed in mice requires homologous recombination (HR) manipulation in embryonic stem (ES) cells (Mansour et al., 1988; Capecchi, 1989). The lack of bona fide germline-competent ES in large animals urged researchers to perform HR in somatic cells rather than in ES cells and then use somatic cell nuclear transfer (SCNT) to produce genetically modified large animals (Polejaeva et al., 2000). Although theoretically possible, the modification of somatic cells by using HR is extremely inefficient; thus, the generation of such cells is impractical (Wells and Prather, 2017). Therefore, only very few gene-targeted pigs were created within two decades (between the establishment of gene targeting technique prior to 1990 and the late 2010s when the novel gene targeting tools began to be used in large animals) (Dai et al., 2002; Lai et al., 2002; Rogers et al., 2008; Suzuki et al., 2012; Davis et al., 2014). This situation changed when newly developed gene targeting technologies called site-specific engineered nucleases or “gene scissors” became available. The designed engineered nucleases, such as zinc finger nucleases (ZFNs), transcription activator-like effector nucleases (TALENs), or clustered regularly interspaced short palindromic repeats (CRISPR)/CRISPR-associated protein (Cas), are very effective in creating double-stranded breaks (DSB) at a specific locus of a genome, thereby facilitating genetic modifications, including knockouts via non-homologous end joining (NHEJ) and knockins via homology-directed repair (HDR) (Hsu et al., 2014) (**Figure 1**). Creation of an intentional DSB in the genomic target can stimulate HR by 50–1000-fold (Jasin, 1996). Therefore, a high rate of DSB formation results in a high rate of modification either in somatic cells or in embryos. With the use of these gene scissors, a large number of genetically modified pigs have been generated through SCNT of modified somatic cells or direct microinjection of engineered nucleases into the embryos. In addition to establishment of genetically modified pigs with agricultural and biomedical values, this technology might have a potentially wider range of application in pigs, such as treatment of viral infections as a therapeutic tool, and gene therapy to correct mutation in pig disease model. These areas still await further investigation.

## Nuclease-Based Gene Editing Tools

### Zinc Finger Nucleases (ZFNs)

Zinc Finger Nucleases are artificial chimeric proteins consisting of a specific DNA-binding domain, which comprises tandem zinc finger-binding motifs, fused to a non-specific cleavage domain of the restriction endonuclease FokI (Kim et al., 1996; Urnov et al., 2010). Zinc finger protein characteristically consists of two beta sheets and an alpha helix, with one or more coordinated zinc ions at their core to confer rigidity to finger (Pavletich and Pabo, 1991). Given that a zinc finger protein unit recognizes 3 bp of DNA, usually in ZFN, 3–6 zinc finger units are combined to recognize 9–18 bp DNA sequences to achieve a specific targeting. By designing two zinc finger motifs recognizing either side of 5–6 bp spacer sequences at a target region, FokI nuclease combined with zinc finger can introduce DSBs within a target region (Kim et al., 1996; Smith et al., 1999; Bibikova et al., 2003; Porteus and Baltimore, 2003; Urnov et al., 2010).

Being the early version of artificial engineered nucleases, ZFN opened a new possibility for gene targeting manipulation in pigs, although this technology still suffers from a complicated construction process and unpredictability of targeting activity. In general, a rational design and assembly of ZFN is somewhat a tough task for many laboratories (Klug, 2010; Lam et al., 2011; Chandrasegaran and Carroll, 2016). An effective ZFN reagent can only be obtained from some commercial sources at a prohibitive price or laboratories that embark in intensive work on ZFN. To the best of my knowledge, studies creating ZFN-mediated genetically modified pigs all use commercially synthesized ZFN reagents. The difficulty of generating active ZFN reagents has impeded their extensive use. Nevertheless, compared with the extremely low gene targeting efficiency of less than 10^-6^ by using conventional HR in somatic cells, ZFN can achieve approximately 1–4% gene targeting rate in selection of modified pig somatic cells (Hauschild et al., 2011; Yang et al., 2011), thereby allowing the cost-effective generation of genetically modified pigs.

### Transcription Activator-Like Effector Nucleases (TALENs)

Transcription activator-like effector nucleases (TALENs) actually have a similar conceptual structure to ZFNs by comprising a DNA binding domain and a DNA cleavage domain and by acting in pairs to satisfy the requirement for dimerization (Christian et al., 2010; Miller et al., 2011). The DNA binding domain of TALENs, named transcription activator-like effector (TALE), originates from the plant-pathogenic bacterium *Xanthomonas* and includes tandem repeat modules of 34 amino acids, with each module specifying the binding to a single base pair. The repeat modules can be rearranged according to a simple cipher to target any DNA sequence (Boch et al., 2009; Moscou and Bogdanove, 2009). Unlike ZFN, TALEN reagents are easy to build by several assembly schemes and can be produced routinely for many laboratories (Cermak et al., 2011; Li et al., 2011; Morbitzer et al., 2011). Aside from their simple design and assembly, TALENs have a broad target range and a substantially improved targeting activity; thus, active TALENs may be designed for almost any DNA target in a genome. About 64% of synthesized TALENs are active in livestock fibroblasts. Three-quarters of these active TALENs demonstrate a high cleavage efficiency (19–40% NHEJ rates). Moreover, TALEN pairs efficiently induce gene knockouts after direct injection of TALEN-encoding mRNA into the cytoplasm of swine and bovine embryos, with a 29% and 43–75% knockout efficiency, respectively (Carlson et al., 2012).

Given that HR-mediated gene knockin results in precise alteration, including point mutation, DNA fragment replacement, and insertion of a new DNA sequence in a target site, knockin modification has a wide range of application prospect compared with the NHEJ-mediated gene knockout, which only causes loss in the functional phenotype of a gene. However, most of the created DSBs were repaired by NHEJ, and only few of them could be repaired by HR when a donor DNA repair template was offered (Mao et al., 2008). Therefore, HR-mediated knockin manipulation is less efficient than NHEJ-mediated knockout even in the presence of engineered nucleases. The high effectiveness of TALEN-mediated gene targeting could achieve an effective gene knockin. Using TALENs and oligonucleotide donor transfection that introduces defined nucleotide changes into multiple targets in the genome of livestock fibroblasts, Tan et al. achieved 10–64% knockin cell colonies, with up to 32% of the homozygous knockin colonies in just one round of transfection (Tan et al., 2013). Another study has established Rosa26 knockin pig models by using TALEN plasmid and long-range arm donor DNA transfection. In this study, the knockin efficiency in selected fibroblasts was as high as 31.3% (60 positive colonies of 192 selected fibroblast colonies) (Li et al., 2014).

### Clustered Regularly Interspaced Short Palindromic Repeats (CRISPR)/CRISPR-Associated Protein (Cas)

Clustered regularly interspaced short palindromic repeats (CRISPR)/CRISPR-associated protein (Cas), originally known as a microbial adaptive immune system, has been adapted for mammalian gene editing recently. The CRISPR/Cas system is based on an adaptive immune mechanism in bacteria and archaea to defend the invasion of foreign genetic elements through DNA or RNA interference (Gasiunas et al., 2012; Jinek et al., 2012; Wiedenheft et al., 2012). Through mammalian codon optimization, CRISPR/Cas has been adapted for precise DNA/RNA targeting and is highly efficient in mammalian cells and embryos. The most commonly used and intensively characterized CRISPR/Cas system for genome editing is the type II CRISPR system from *Streptococcus pyogenes*; this system uses a combination of Cas9 nuclease and a short guide RNA (gRNA) to target specific DNA sequences for cleavage. A 20-nucleotide gRNA complementary to the target DNA that lies immediately 5′ of a PAM sequence (NGG) directs Cas9 to the target DNA and mediates cleavage of double-stranded DNA to form a DSB (Cong et al., 2013; Mali et al., 2013). Thus, CRISPR/Cas9 can achieve gene targeting in any N20-NGG site.

Since CRISPR/Cas9 first emerged, researchers have been highly impressed by its incomparable gene targeting efficiency and simple construction of customized vectors compared with previous site-specific nucleases. These characteristics render CRISPR/Cas9 highly accessible for almost any laboratories, thereby significantly contributing to the progress of research in many areas of biomedicine and agriculture. The high cleavage activity of CRISPR/Cas9 allows simultaneous targeting of multiple loci in a single cell within a single reaction (Cong et al., 2013). GGTA1/iGb3S and GGTA1/CMAH double knockout and GGTA1/iGb3S/CMAH triple knockout pigs, which have potential for xenotransplantation, were created by a single-step transfection of multiplexed sgRNA and Cas9 nuclease together with a single nuclear transfer (Li et al., 2015). We generated homozygous Pink1/Parkin double knockout pigs as Parkinson’s disease models through a single transfection of CRISPR/Cas9 and SCNT. The frequency of selecting homozygous double knockout fibroblast colonies could reach up to 38.1% (Zhou et al., 2015).

## Genetically Modified Pigs for Agricultural Application

Traditional selective breeding has produced a series of superior livestock varieties that demonstrate a dramatically enhanced production performance compared with their original counterparts. However, individual trait shows only 0.5–3.0% genetic response to selection per year, and some traits such as fertility and disease resistance remain difficult to improve (Clark and Whitelaw, 2003). Furthermore, when production traits have been improved to a certain degree, their further optimization would even be more difficult and would need an even longer breeding cycle to achieve even a slight progress. Genome editing offers an alternative approach to rapidly and directly realize genetic improvement in livestock (Ruan et al., 2017). Fully improvement of individual and even multiple traits can be accomplished within only one generation. Importantly, by using genome editing, we can confer upon animals favorable genetic traits that are unavailable in natural genetic sources, thereby generating novel livestock varieties that cannot be achieved through traditional breeding.

### Meat Production

Myostatin (MSTN) is a negative regulating factor of skeletal muscle mass *in vivo*. MSTN knockout mice exhibit a two–threefold increase in muscle mass due to muscle fiber hyperplasia and hypertrophy (McPherron et al., 1997). Natural mutations of MSTN have been found in several species, including cattle (Grobet et al., 1997; Kambadur et al., 1997; McPherron and Lee, 1997), sheep (Clop et al., 2006), and dogs (Mosher et al., 2007). MSTN modification is an effective approach to enhance muscle growth in various animals. Given that this gene is a “hot” candidate that is possibly beneficial in agriculture, several strains of MSTN-knockout pigs have been generated by using ZFNs (Qian et al., 2015), TALENs (Rao et al., 2016), and CRISPR/Cas9 (Wang et al., 2015, 2017b; Bi et al., 2016). These MSTN-knockout pigs demonstrate muscle hypertrophy or double-muscled (DM) phenotype, with increased muscle mass and decreased fat accumulation compared with wild-type (WT) pigs. One study showed some MSTN-mutant pigs with one extra thoracic vertebra (Qian et al., 2015). MSTN-knockout pigs are valuable breeding materials for rapid genetic improvement to produce lean meat from fat-type (indigenous) pig breeds.

However, some deleterious effects were found in some MSTN-null DM pigs. In homozygous MSTN knockout piglets of the Landrace breed, newborns have abnormal forelegs and/or hind legs and thus their motor function is severely impaired. The affected piglets usually die quickly after birth (Zou et al., 2018). MSTN possibly plays an important role in the development and function of muscles and other organs as MSTN is expressed from early embryogenesis through adulthood. It seems that MSTN modification could result in severe side effect in some pig breeds, albeit not observed in all reported MSTN-knockout pigs. Therefore, a careful selection of pig breeds which show an increased meat production but minimized collateral damage by MSTN modification is needed for establishment of MSTN-knockout DM pig breeds. A new candidate target (FBXO40) that influences muscle production has been found recently. FBXO40 knockout pigs display a muscle hypertrophy phenotype and survive normally without detectable pathological changes in major organs (Zou et al., 2018). Also of note is that the DM animals are more susceptible to respiratory disease, lameness, stress and dystocia, thus requiring extra attention in husbandry to preserve animal welfare (Fiems, 2012).

### Viral Resistance

Porcine reproductive and respiratory syndrome virus (PRRSV) is the most economically important swine disease worldwide, currently causing huge economic losses in the swine industry. Genome editing shed light on the establishment of PRRSV-resistant pigs through knockout of viral receptors in pigs. Potential PRRSV entry mediators, SIGLEC1 and CD163 were knocked out in pigs through conventional HR and CRISPR/Cas9, respectively (Prather et al., 2013; Whitworth et al., 2014). PRRSV challenge in knockout pigs demonstrated that SIGLEC1 is unnecessary for infectivity (Prather et al., 2013), whereas CD163 is the definitive receptor for PRRSV. CD163 knockout pigs are fully resistant to PRRSV challenge with no obvious PRRSV-related symptom and no detectable PRRSV antibody and RNA in the serum (Whitworth et al., 2016). Furthermore, multiple genotypes of CD163 modifications were achieved by using CRISPR/Cas9 in different laboratories; these modifications include CD163 total knockout (CD163 null) (Yang et al., 2018), domain swap of CD163 exon 7 (corresponding to SRCR 5, the PRRSV binding domain at the protein level) with human CD163L-1 exon 11 (chimeric CD163) (Wells et al., 2017), and CD163 truncation with a deletion of exon 7 (ΔSRCR5 CD163) (Burkard et al., 2017). Among these CD163-modified pigs, those with CD163 null phenotype were completely resistant to both Type 1 and Type 2 PRRSV isolates (Wells et al., 2017). The chimeric CD163 phenotype was resistant to Type 1 PRRSV but still supported the replication of the Type 2 virus (Wells et al., 2017). Our group also generated CD163 knockout pigs with null phenotype, and they demonstrated their complete resistance to the highly pathogenic PRRSV, which is the dominant circulating strain in China and other Asian countries (Yang et al., 2018).

### Thermoregulation

Due to their lack of a functional UCP1 gene, pigs lack brown adipose tissue (BAT). As a result, the BAT-mediated adaptive non-shivering thermogenesis is absent in pigs (Trayhurn et al., 1989; Berg et al., 2006; Jastroch and Andersson, 2015). Newborn piglets are thus susceptible to cold stress, which may result in neonatal death. To address this issue, Zheng et al. inserted a mouse adiponectin-driven UCP1 into the porcine endogenous UCP1 locus by using a CRISPR/Cas9-mediated knockin strategy combined with SCNT. The resultant UCP1 knockin pigs showed an improved ability to maintain body temperature when acutely exposed to cold. Moreover, UCP1 prevented obesity by reducing fat deposition, an economically important trait targeted in pig breeding. UCP1 knockin pigs demonstrated reduced fat deposition through UCP1-promoted lipolysis (Zheng et al., 2017). Thus, UCP1 knockin pigs are a potentially valuable genetic resource for agricultural production on the basis of their improved thermoregulation and decreased fat deposition.

### Recipients for Spermatogonial Stem Cell (SSC) Transplantation

Spermatogonial stem cells sustain normal spermatogenesis and maintain male fertility through self-renewal and differentiation. SSCs can be transplanted to generate donor-derived offspring (Brinster and Zimmermann, 1994). From the agricultural perspective, SSC transplantation is a potential tool to rapidly expand the availability of gametes from desirable superior livestock, thereby dramatically influencing production efficiency, quality, and other production traits in a population (Ehmcke et al., 2006). A recipient male lacking endogenous SSC and other germ cells but preserving intact somatic support cells is required for a successful SSC transplantation. Although chemotoxic drug or irradiation was employed to destroy spermatogenesis and cause infertility, the outcome was not ideal as manifested by the incomplete elimination of endogenous germ cells or the severe side effect on the recipient animals (Oatley, 2017; Park et al., 2017). An approach to eliminate SSC by knockout of the gene essential for SSC development has established an ideal surrogate for SSC transplantation. Park et al. generated NANOS2 knockout pigs by directly injecting CRISPR reagents into the cytoplasm of embryos. Knockout males could not produce sperm but still kept intact seminiferous tubules structure, thus had the potential to serve as an ideal SSC recipient (Park et al., 2017).

## Genetically Modified Pigs as Disease Models

Genome editing has extensively and intensively promoted the application of pigs as human disease models. Changing the pig genome allows these animals to resemble the mutations causing genetic disorder in humans, and pigs could phenocopy human disease manifestations more accurately than the commonly used mouse models. The suitable size and long lifespan of pig disease models also facilitates carrying out surgical manipulation closer to clinical conditions and long-term tracking and evaluation of therapeutics over clinically relevant time frames. Using the genome editing tools, genomic changes could occur not only in a single gene but also in multiple genes simultaneously with a high editing efficiency, thereby paving a way to mimic and decipher complex polygenic heredity diseases in large animal models similar to humans.

### Neurodegenerative Diseases

Genetically modified pigs have been successfully used to establish animal models of neurodegenerative diseases, including Huntington’s disease (HD) and Parkinson’s disease (PD) (Zhou et al., 2015; Yan et al., 2018). Yan et al. reported a huntingtin (HTT) knockin pig as Huntington’s disease model, in which pig HTT exon 1 containing 18 CAG repeats was replaced with human HTT exon 1 containing a 150-CAG repeat with CRISPR/Cas9. An expanded CAG repeat causes the HD phenotype (Mangiarini et al., 1996). The cloned HTT knockin pigs showed less weight gain compared with age- and sex-matched WT pigs, and HD-like symptoms, including deficient motor function and respiratory difficulty usually observed in HD patients. Morphological analysis revealed neuropil and nuclear HTT aggregates in the brain of knockin pigs, as well as a marked decrease in the number of neurons in the striatum compared with that in the cortex and cerebellum; this condition is similar to the selective neurodegeneration in the striatum of brains of HD patients (Yan et al., 2018).

Parkinson’s disease is the second most common form of neurodegenerative disorders. Approximately 10% of PD are familiar cases with many disease-associated/caused genetic mutations were identified (Lesage and Brice, 2009). Zhou et al. (2015) reported on Pink1/Parkin double knockout pigs generated using CRISPR/Cas9 in a one-step transfection and cloning. The brains of cloned knockin pigs lost the expression of Pink1 and Parkin, but no PD-associated phenotypic changes were found (Zhou et al., 2015). These pigs are expected to show late onset of the disease because PD is a progressive disease with a mean onset at around the age of 60 in humans.

### Cardiovascular Diseases

Cardiovascular diseases are the number one cause of death and disability worldwide (Joseph et al., 2017). As the most commonly used disease models, mice usually cannot accurately model the physiology and pathology of the human cardiovascular system given their significantly different heart size and rate compared with humans (Milani-Nejad and Janssen, 2014). Therefore, mouse models usually fail to predict human outcomes. For example, thiazolidinediones (TZDs), selective ligands of PPAR-γ, can sensitize insulin to treat type 2 diabetes. Although TZD-mediated activation of PPAR-γ exerts a beneficial effect on cardiovascular diseases in mouse models, the therapeutic efficacy of TZDs has been severely compromised because of the increased risk of adverse cardiovascular events in patients (Nissen and Wolski, 2007). Pig and human myocardia are highly similar. Thus, pigs are the most attractive models bridging the gap between humans and mice. Yang et al. (2011) established PPAR-γ heterozygous knockout pigs by using ZFN. Their work was the first to use the genome editing tool to knockout an endogenous gene of large animals (Yang et al., 2011).

The loss of function of low-density lipoprotein receptor (LDLR) and Apolipoprotein E (ApoE) has been implicated in the progression of atherosclerosis, the primary culprit for cardiovascular diseases (Mahley, 1988; Hasler-Rapacz et al., 1998; Sehayek et al., 2000; Rader et al., 2003). Given that the lipoprotein profiles and metabolism in mice differ from those in humans, atherosclerosis pig models may benefit atherosclerosis research. ApoE/LDLR double knockout pigs have been recently established by using CRISPR/Cas9 (Huang et al., 2017). These pigs show an abnormal lipid metabolism related to atherosclerosis. Prior to that, several strains of LDLR knockout pigs, including Yucatan miniature pigs engineered using the traditional HR (Davis et al., 2014) and Ossabaw pigs engineered by using TALENs (Carlson et al., 2012), were generated. Among the models, LDLR knockout Yucatan miniature pigs, which are used to model atherosclerosis, have been well characterized. LDLR-deficient pigs had considerably elevated levels in total and LDL cholesterol fed a standard diet, resulting in atherosclerotic lesions in the coronary arteries and abdominal aorta that resemble human familial hypercholesterolemia and atherosclerosis in a short time (Davis et al., 2014). Recently, LDLR-deficient Yucatan pigs were used to test the effect of bempedoic acid on lowering LDL cholesterol and in attenuating atherosclerosis, indicating the value of LDLR-deficient pigs in preclinical evaluation of therapeutics (Burke et al., 2018).

### Cancer

Acquired mutations are the most common causes of cancer. Instability of genome, activation of oncogenes, and inactivation of tumor-suppressor genes all can result in different types of cancer. An optimal cancer animal model should be established by inducing genetically defined tumors in a tissue-specific manner rather than by genetically engineering the animal. Wang et al. (2017a) established a Cre-dependent inducible Cas9-expression pig with CRIPSR/Cas9-mediated knockin in the Rosa26 locus. This pig model allows *ex vivo* genome editing in isolated pig cells and *in vivo* genome editing by introducing a corresponding gRNA. To mimic an abnormal oncogenic EML4-ALK fusion gene identified in a subset of non-small-cell lung cancers (NSCLCs) (Soda et al., 2007), two gRNAs targeting EML4 and ALK were introduced into the isolated pig fibroblasts via lentivirus. Fibroblasts with oncogenic EML4-ALK fusion gene arising through a paired CRISPR/Cas9-mediated genome inversion could be generated. Moreover, the study investigated the feasibility of lung cancer induction *in vivo* through intranasal delivery of multiplexed gRNAs targeting tumor suppressor genes TP53, PTEN, APC, BRCA1, and BRCA2, as well as oncogene KRAS. Three months after gRNA administration, the Cre-dependent Cas9-expressing pigs presented signs of pneumonopathy, and morphological analysis of lung tissue showed a pathological feature similar to the human adenocarcinoma. The target genes harbored insertions/deletions close to the CRISPR-cleavage site, indicating a loss-of-function mutation in the tumor suppressor genes. For the oncogene KRAS, gain-of-function mutations induced by CRISPR/Cas9 were observed, with genotypes similar to that in the potent oncogenic mutations in human lung tumors (Wang et al., 2017a). The inducible Cas9 pig models offer an ideal platform for inducing tumor-associated somatic mutations *in situ* to model human cancer. Apart from this model, several other pig cancer models were produced, including knockout of tumor suppressors P53 and RUNX3 by TALENs and CRISPR/Cas9 as the germline mutations, respectively (Kang et al., 2016; Shen et al., 2017). The carcinogenic phenotypes of these animals require further investigation.

### Immunodeficiency

An animal with severe combined immune deficiency (SCID) not only mimics human diseases but also serves as a valuable research tool for cancer, stem cell, cell therapy, and organ transplantation. The interleukin-2 receptor gamma (IL2RG) knockout pigs that were generated using conventional HR or ZFN exhibited X-linked SCID, in which T and NK cells were absent (Suzuki et al., 2012; Watanabe et al., 2013). In generation of ZFN-mediated IL2RG knockout pigs, ZFN-encoding mRNA was transfected into male porcine fibroblasts to target IL2RG. The researchers obtained 1 ZFN-induced knockout cell line from 192 single cell-derived cell lines obtained by limiting dilution (0.5% targeting efficiency) (Watanabe et al., 2013). In the allogeneic bone marrow transplantation, the lymphoid lineage of the SCID pigs was reconstituted by donor cells and survival time was prolonged through restoration of immune function (Suzuki et al., 2012). This SCID pig serves as an essential preclinical model to evaluate stem cell therapy. In 2014, two laboratories separately established RAG1/2 knockout pigs by using TALENs (Huang et al., 2014; Lee et al., 2014). RAG knockout pigs exhibited an SCID phenotype and lacked T and B cells. In one study, human induced pluripotent cells were injected into these SCID pigs, which developed teratomas that represent a wide range of human tissues.

In another study, B cell-deficient pigs were generated by applying CRISPR/Cas9 to target the IgM heavy chain gene, which is crucial in B cell development and differentiation (Chen et al., 2015). The cloned modified pigs manifested a depletion of both B cells and antibody in their blood and thus can be used to model human B cell deficiency. Moreover, the pig models can be further engineered for large-scale production of therapeutic humanized polyclonal antibodies for clinical use.

## Genetically Modified Pigs for Xenotransplantation

Domestic pigs are the most suitable donors for xenotransplantation to alleviate the growing shortage of allogeneic donor organs for clinical transplantation to treat patients with end-stage organ failure. Pigs can provide size-matched organs at an affordable cost. However, the high immune incompatibility between the donor and the recipient is the major barrier for xenotransplantation. The immunological hurdles to xenotransplantation include but not limited to hyperacute rejection, delayed xenograft rejection, acute cellular rejection, and chronic rejection (Yang and Sykes, 2007; Hryhorowicz et al., 2017). Advances in genome editing enable genetic modifications in pigs to reduce cross-species immune barrier and prevent xenograft rejection.

The host normally destroys xenografts within minutes or hours through hyperacute xenograft rejection, which inevitably leads to failure. The main reason for hyperacute rejection of xenograft is the presence of naturally occurring antibodies in human plasma that recognizes the Galactose α(1–3)Galactose (Galα(1–3)Gal) antigen on the surface of porcine endothelial cells. Synthesis of Galα(1–3)Gal is catalyzed by the enzyme a-1,3-galactosyltransferase (GGTA1), which is present in pigs but absent in humans (Good et al., 1992; Galili, 1993). As early as 2002, pigs with heterozygously knockout GGTA1 were produced using HR (Dai et al., 2002; Lai et al., 2002), and homozygous GGTA1 knockout (GTKO) piglets were born by further screening of homozygous knockout cells from the heterozygous pigs (Phelps et al., 2003; Kolber-Simonds et al., 2014). Heart xenotransplantation from GTKO pigs into immune-suppressed baboons showed a mean graft survival period of 99 days, and the longest surviving graft functioned in the recipient for 179 days (Kuwaki et al., 2005). Generation of GTKO pigs indicates that hyperacute xenograft rejection has been overcome to a great extent. A series of GTKO pigs with different genetic backgrounds has been recently established by using genome editing tools (Hauschild et al., 2011; Xin et al., 2013; Bao et al., 2014; Feng et al., 2016; Petersen et al., 2016; Chuang et al., 2017). Furthermore, other xenoreactive antigens present in pigs but absent in humans have been identified; these antigens include Neu5Gc antigen (*N*-glycolylneuraminic acid) catalyzed by cytidine monophosphate-*N*-acetylneuraminic acid hydroxylase (CMAH) and a glycan produced by β1,4-*N*-acetylgalactosaminyltransferase (B4GALNT2) (Byrne et al., 2015, 2018). In addition, corresponding knockout pigs with double (GGTA1/CMAH) and triple (GGTA1/CMAH/B4GALNT2) gene inactivation were established (Lutz et al., 2013; Estrada et al., 2015; Miyagawa et al., 2015; Gao et al., 2017). Based on the GTKO pigs, numerous other genetic modifications were performed to further overcome xenogeneic barriers; these approaches mainly include individual or combined overexpression of transgenes, such as CD46, CD55, CD59, CD39, thrombomodulin, heme oxygenase 1, A20, HLA-E, and CD47, to prevent complement activity, delayed xenograft rejection, or/and acute cellular rejection (Fischer et al., 2016; Hryhorowicz et al., 2017; Laird et al., 2017). These pigs were produced through transgenesis and thus excluded in this review for a deep discussion. These multi-modified pigs may exhibit further immune tolerance to attenuate xenograft injury.

Besides the concern on pig-to-human immune barrier, another notable issue in xenotransplant is the risk of cross-species transmission of porcine endogenous retroviruses (PERVs), which are dormant (inactive) endogenous retroviruses constituting an integral part of the porcine genome; PERVs may be reactivated by certain factors or changes in the environment and thus become infectious (Patience et al., 1997; van der Laan et al., 2000). PERVs in xenografts possess the potential to become pathogenic and infectious in the recipient. Elimination of PERV in porcine genome is difficult because they are integrated in multiple locations in the genome. Yang et al. (2015) used CRISPR/Cas9 to disrupt all 62 copies of the PERV pol gene in the immortal pig cell line PK15; by using the PERV knockout cells, they demonstrated a >1000-fold reduction in PERV transmission to human cells (Yang et al., 2015). The same group has recently cloned PERV inactivated pigs combining CRISPR/Cas9 and SCNT, in which all 25 copies of functional PERVs were inactivated (Niu et al., 2017). The use of PERV knockout pigs addressed the safety concern in clinical xenotransplantation. Moreover, the powerful ability of the CRIPSR tool to target dozens of genomic sites simultaneously provides infinite possibility to create animals harboring complex modifications.

In this section, we present an emerging cutting-edge technology, that is, growing of humanized organ in pigs or called xeno-generation; this technology is greatly promoted with the use of genome editing. Compared with directly modifying pig as an organ donor, xeno-generation includes interspecies blastocyst complementation combining donor (human) pluripotent stem cells and organogenesis-disabled hosts (pigs), allowing the enrichment of donor cells in a target organ to form a chimeric animal harboring a humanized target organ, which can be finally used for transplantation (Wu et al., 2016). In this regard, organogenesis-disabled pigs can be simply realized by genome editing when the gene controlling the development of the target organ is identified. Matsunari et al. (2013) demonstrated the feasibility of isogeneic organ generation using blastocyst complementation in pigs. In which, the transgene Hes1 under the Pdx1 promoter (Pdx1-Hes1) was used to suppress pancreatic development, resulting in the creation of apancreatic pigs compatible for pancreatogenesis derived from donor cells. Wu et al. (2017a) reported on an interspecies chimerism that human pluripotent stem cells could integrate and differentiate in a pig embryo, constituting a big step toward xenogeneic organ generation. Furthermore, this group disabled pancreatogenesis in pigs through knockout of PDX1 using CRISPR/Cas9, creating a suitable platform for realizing human organogenesis in pig (Wu et al., 2017b).

## Conclusive Thoughts

### Comparison of Three Engineered Nucleases

Engineered nuclease-based genome editing has revolutionized the creation of genetically modified pigs, thus expanding their utilization in diverse research fields. As the prelude of next-generation genome editing technology, ZFN surprised researchers for opening an effective approach to modify a target genome site. The gene targeting efficiency of ZFN, although limited, remains considerably higher than that of traditional HR. TALEN quickly overtook ZFN technology when it first appeared due to its higher efficiency in gene targeting, greater flexibility in targeting specific sequences, and ease of construction. Moreover, the emergence of CRISPR/Cas system represents a major leap for remarkably efficient specific genetic modification in mammalian cells and zygotes. In addition, the design and production of gRNA are through a quick and simple procedure by the *in vitro* transcription of synthetic DNA oligonucleotides or by the cloning of oligonucleotides into expression vectors; this process offers a clear advantage over the production of ZFN and TALEN. However, few concerns emerged in CRISPR/Cas, such as requirement of a PAM sequence adjacent to the 3′ end of the target sequence and a high frequency of off-target cleavage. The requirement of a specific PAM sequence often restricts the range of targetable sequences. The commonly used Cas9 protein derived from *S. pyogenes* utilizes NGG as the PAM sequence, but recent exploitation of Cas9 orthologs from other bacterial species or redesigned/evolved Cas9 can recognize different PAM sequences, thereby increasing flexibility in genome editing. A study reported on the fusion of Cas9 with a programmable DNA-binding domain at an improved precision and increased targeting range. The Cas9 fusion protein was equally efficient for a range of PAMs, including NAG, NGA, NGC, and NGG (Bolukbasi et al., 2015). Another study used phage-assisted continuous evolution to generate an expanded PAM SpCas9 variant that can recognize a broad range of PAM sequences, including NG, GAA, and GAT. Moreover, the SpCas9 variant demonstrated a considerably greater DNA specificity than the original SpCas9 (Hu et al., 2018). The current Cas9 family expands the DNA targeting scope of CRISPR systems and is suitable at nearly any genomic locus. With regard to off-target, a typical TALEN target sequence usually covers 30 nt, which is unique within the genome, whereas CRISPR/Cas recognizes 20 nt target and can allow multiple mismatches in the guide sequence, thereby increasing the likelihood of off-target effects (Fu et al., 2013; Hsu et al., 2013). A possible solution is the use of Cas-nickases guided by a pair of gRNAs targeting the opposite strands for cooperative genome editing, as the longer target site increases the on-target precision (Mali et al., 2013; Ran et al., 2013). In addition, various strategies have been developed to improve the targeting specificity; these strategies include optimized design of gRNA, Cas9 enzyme engineering, and off-target detection assays (Tycko et al., 2016). Notably, two recent engineered enzymes (eSpCas9 and SpCas9-HF) have elegantly increased SpCas9 specificity by reducing tolerance for mismatched DNA binding (Kleinstiver et al., 2016; Slaymaker et al., 2016).

### Approaches in Pig Genome Editing: SCNT Versus Embryo Injections

Somatic cell nuclear transfer or cloning involves screening of somatic cells (typically fetal fibroblasts), which carry the intended genetic alterations, and the nuclear transfer of the modified cells in a cloning process. Engineered nuclease can be easily applied to create either NHEJ- or HDR-induced mutations within a donor cell *in vitro* through a pre-screening or selection strategy, which enables enrichment of cells carrying the desired mutation (Zhou et al., 2015). An alternative to SCNT is the method involving direct gene editing in single-cell embryos. The mRNA of editors (for knockout) or together with donor DNA (for knockin) can be microinjected into the cytoplasm or pronucleus of zygotes, which are then transferred into the synchronized surrogates to generate edited animals. This procedure is vastly simple compared with SCNT (Lillico et al., 2013).

The major advantage of SCNT over direct embryo injection is the predictable genotype of the founder pigs. By contrast, pigs generated via embryo injection usually contain mosaic genotypes with multiple modification types in different cells, and several cycles of breeding are usually needed to produce homozygously modified pigs with identical genotype. In some situations, the chimeric founder includes intact WT germ cells and thus cannot generate genetically modified progeny. However, SCNT of genome-edited cells suffer from an impaired embryonic development probably due to the off-target effect or other unidentified toxicity of the genome editing nucleases. Therefore, genome-edited somatic cells usually have a relatively lower cloning efficiency than WT and randomly integrated transgenic cells. Taking into account the aspects mentioned above, embryos injection is currently the preferable ways for production of CRISPR/Cas9-edited pigs in many laboratories. The extremely high editing activity and low toxicity to embryos during microinjection of CRISPR/Cas9 mRNA cocktail can reduce mosaicism and even generate homozygously knockout founder pigs with identical mutation (Whitworth et al., 2017). However, knockin manipulation remains a limitation because few knockin pigs generated through zygote injection have been reported to date (Peng et al., 2015; Zhou et al., 2016). The field still awaits for the optimal strategies that enhance HDR, such as drug treatment, optimal design of donor, and new generation of editing tools.

### Other Emerging Genome Editing Nucleases

The number of reported genetically unique large animals dramatically increases as a result of the extensive use of genome editing tools. Moreover, the types of endonucleases used in genome editing are rapidly increasing. In the Cas9 system, many Cas9-like nucleases were developed given the natural diversity of bacterial CRISPR systems. Cpf1, a putative Class 2 CRISPR effector, mediates target DNA editing with distinct features from Cas9 (Zetsche et al., 2015). In contrast to Cas9 which generates blunt ends, Cpf1 generates a 5-nt staggered cut with a 5′ overhang, which is particularly advantageous in facilitating a NHEJ-based gene insertion (knockin) into a genome. Recently, the CRSIPR/Cpf1-mediated dystrophin knock-out pigs, and phospholamban knock-in pigs with a 3-nt deletion in the presence of a single-stranded oligo donor, have been established as a Duchenne muscular dystrophy (DMD) and dilated cardiomyopathy (DCM) models, respectively. In this study, the CRSIPR/Cpf1 induced 41.8% knockout rate and 2% knockin rate in the selected fibroblast colonies (Wu et al., 2018). A hybrid enzyme combining the Cas9-nickase and PmCDA1, an activation-induced cytidine deaminase could perform targeted nucleotide substitution (C-U) without the use of template DNA, providing a novel route for point mutation (Nishida et al., 2016). A CRISPR system (Cas13a) that targets RNA has also been developed recently (Abudayyeh et al., 2017). In addition to the CRISPR system, Xu et al. (2016) designed a structure-guided endonuclease (SGN) consisting of flap endonuclease-1 that recognizes the 3′ flap structure and the cleavage domain of Fok I, which cleaves DNA strands. A guide DNA complementary to the target with an unpaired 3′ end is needed to form a 3′ flap structure. SGN recognizes and cleaves the target DNA on the basis of the 3′ flap structure of a double-flap complex formed between the target and the guide DNA. The SGN offers a strategy for a structure-based recognition, capture, and editing of any desired target DNA, thereby expanding the toolkit for genetic modification (Xu et al., 2016). Taken together, these systems are expected to substantially broaden the application of artificial engineered nucleases and facilitate the establishment of excellent pig models with desirable genotypes in agriculture and biomedicine.

## Author Contributions

HY wrote the initial draft of the manuscript and worked on subsequent revisions. ZW worked on revising the manuscript.

## Conflict of Interest Statement

The authors declare that the research was conducted in the absence of any commercial or financial relationships that could be construed as a potential conflict of interest.
